# Prognostic Nutritional Index Could Serve as a Reliable Prognostic Marker in Intensive Care Population

**DOI:** 10.3390/medsci13020059

**Published:** 2025-05-11

**Authors:** Ibrahim Karagoz, Songul Peltek Ozer, Bahri Ozer, Gulali Aktas

**Affiliations:** 1 Anesthesiology & Reanimation Deptartment, Abant Izzet Baysal University Hospital, 14030 Bolu, Turkey; dr.ikar@hotmail.com; 2 Pathology Department, Abant Izzet Baysal University Hospital, 14030 Bolu, Turkey; songulpeltek@hotmail.com; 3 General Surgery Department, Abant Izzet Baysal University Hospital, 14030 Bolu, Turkey; bahriozer@hotmail.com; 4 Internal Medicine Department, Abant Izzet Baysal University Hospital, 14030 Bolu, Turkey

**Keywords:** prognostic nutritional index, inflammation, mortality, intensive care unit

## Abstract

**Background**: Morbidity and mortality rates in intensive care units (ICUs) reflect the severe health challenges faced by critically ill patients. Nutritional and immune status, as measured by the prognostic nutritional index (PNI), are increasingly recognized as important predictors of intensive care unit outcomes. **Objective**: We aimed to compare the prognostic nutritional index levels of survived and deceased subjects treated in intensive care units. **Methods**: This retrospective study examined the association between prognostic nutritional index and mortality among intensive care unit patients treated from June 2023 to June 2024. The prognostic nutritional index was calculated using serum albumin and lymphocyte levels, and patients were categorized into survived and deceased groups. Statistical analyses, including ROC and logistic regression, were used to evaluate prognostic nutritional index’s predictive capacity. **Results**: We revealed that deceased patients had significantly lower prognostic nutritional index values, lower platelet counts, and higher C-reactive protein (CRP) and serum creatinine levels compared to survivors. The prognostic nutritional index was independently associated with mortality, with each unit increase decreasing mortality risk by 6%. **Conclusion**: These findings highlight the prognostic nutritional index’s utility as a prognostic tool in intensive care unit settings, underscoring the need for nutritional assessments and targeted interventions to improve patient outcomes. Further research with larger cohorts is warranted to validate these findings and explore causative mechanisms.

## 1. Introduction

Morbidity and mortality in intensive care units (ICUs) are key indicators of patient outcomes in critical care settings. The accurate prediction of these outcomes is crucial, and several scoring systems have been developed for this purpose [1]. Additionally, inflammation is a common characteristic among ICU patients, and inflammatory markers have been found to be associated with mortality in this population [2]. Nutritional indices are also used to assess poor nutritional status, which is linked to a higher risk of infections, delayed wound healing, increased complications, and prolonged hospital stays [3]. The prognostic nutritional index (PNI) is one such tool, designed to evaluate a patient’s nutritional and immunological status, particularly in surgical or critically ill patients [4]. It is calculated using serum albumin levels and total lymphocyte count, offering a simple yet effective method for assessing overall health and recovery potential. The PNI is frequently used to estimate the risk of complications, morbidity, and mortality in surgical patients and those with cancer [5,6,7,8]. By identifying at-risk individuals early, the PNI can help guide nutritional interventions and other supportive measures aimed at improving outcomes and reducing postoperative complications [9]. Nutritional indices have been investigated as predictors of mortality in ICU populations [10]. The Controlling Nutritional Status (CONUT) score has been recommended as a prognostic tool for hospitalized patients [11]. Additionally, both the PNI and CONUT scores have been shown to correlate with the prognosis of patients with acute heart failure [12].

In the present retrospective analysis, we hypothesized that the prognostic nutritional index may be associated with mortality in patients treated in intensive care units. Therefore, the objective of this study was to evaluate the prognostic nutritional index values in an ICU population by comparing prognostic nutritional index levels between survivors and non-survivors.

## 2. Materials and Methods

### 2.1. Study Population

**Figure 1:**
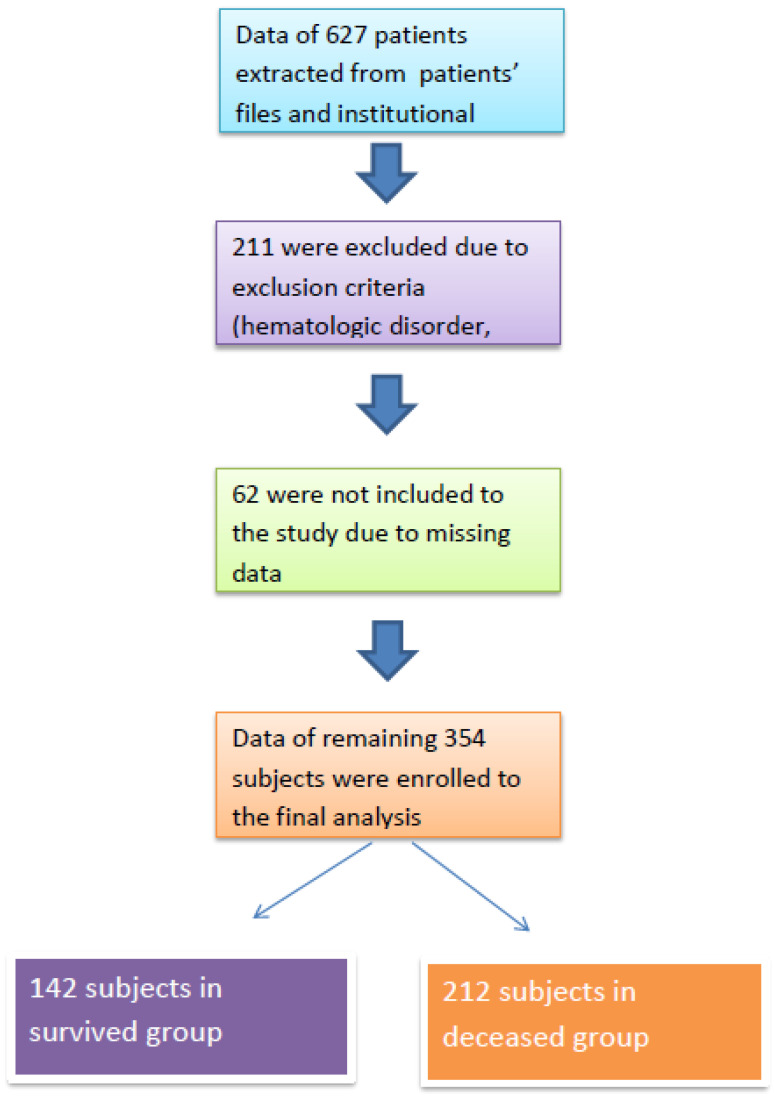
Study flow chart.

After obtaining approval from the institutional review board (Date: 4 June 2024; Approval No: 2024/142), we retrospectively analyzed patients who were treated in the intensive care unit of our institution between June 2023 and June 2024. Patients who required ICU treatment due to infection or acute complications of chronic conditions (e.g., chronic obstructive pulmonary disease, heart failure, diabetes mellitus, chronic kidney disease) were included in the study. Patients with acute trauma, those under 18 years of age, those who received human albumin therapy, and those with hematologic disorders or malignant conditions were excluded. A flow chart of the study population is presented in Figure 1.

Data on general characteristics, including age, gender, and ICU length of stay, and laboratory results were collected from patient records and the institutional database. Laboratory parameters included leukocyte count (WBC), neutrophil count (NEU), lymphocyte count (LYM), monocyte count (MONO), hemoglobin (Hb), hematocrit (Hct), red cell distribution width (RDW), platelet count (PLT), platelet distribution width (PDW), mean platelet volume (MPV), plasma glucose, serum creatinine, CRP, and serum albumin. All biochemical assays were conducted with an automatic analyzer (Architect c 8000, Abbott Laboratories, Chicago, IL, USA) following the manufacturer’s instructions. All laboratory values were obtained from the initial assessment upon ICU admission. The PNI was calculated using the following formula: (10 × serum albumin [g/dL] + (0.005 × blood lymphocyte count [×10^3^/mm^3^]) [3]. Patients who were discharged from the ICU to the general ward were classified as survivors, while those who died during their ICU stay were classified as non-survivors. Data from the survivor and non-survivor groups were compared.

### 2.2. Statistical Analyses

Statistical analyses were performed using SPSS version 16.0 for Windows (IBM Corp., Chicago, IL, USA). The normality of data distribution was assessed using the Kolmogorov–Smirnov test. Variables with normal distribution were expressed as mean ± standard deviation and compared using the independent samples t-test. Non-normally distributed variables were expressed as median and interquartile range (IQR) and compared using the Mann–Whitney U test. Categorical variables were expressed as numbers and percentages and were compared using the chi-square test. A Pearson correlation analysis was used to examine associations between study variables. The sensitivity and specificity of each variable in predicting mortality were assessed using a receiver operating characteristic (ROC) curve analysis. Binary logistic regression was performed to evaluate whether the PNI was an independent predictor of mortality, adjusting for age, mean platelet volume, PLT, CRP, and serum creatinine. A *p*-value of <0.05 was considered statistically significant.

## 3. Results

A total of 354 subjects were included in the final analysis, with 142 in the survivor group and 212 in the non-survivor group. The median ages of the survivor and non-survivor groups were 68 (IQR: 27) and 73 (IQR: 22) years, respectively (*p* < 0.001).

Eighty-two (58%) of the survivors and 130 (61%) of the non-survivors were male. There was no statistically significant differences in gender distribution between the groups (*p* = 0.50).

No significant differences were observed between survivors and non-survivors in terms of hemoglobin (*p* = 0.48), hematocrit (*p* = 0.56), white blood cell count (*p* = 0.46), neutrophil count (*p* = 0.19), monocyte count (*p* = 0.30), red cell distribution width (*p* = 0.40), platelet distribution width (*p* = 0.09), or plasma glucose levels (*p* = 0.06). Table 1 summarizes the general characteristics and laboratory data of the study population.

The median albumin levels were significantly higher in survivors (3.2 [0.8] g/dL) compared to non-survivors (2.8 [0.7] g/dL; *p* < 0.001). Median lymphocyte counts were also higher in survivors (1.12 [0.8] × 10^3^/mm^3^) than in non-survivors (1.00 [0.9] × 10^3^/mm^3^; *p* = 0.02). Survivors had significantly higher platelet counts (217 (115) ×10^3^/mm^3^) compared to non-survivors (199 (157) × 10^3^/mm^3^; *p* = 0.002). Similarly, the mean platelet volume was significantly higher in the survivor group (9 (3) fL) than in the non-survivor group (8 [2.9] fL; *p* < 0.001).

Conversely, C-reactive protein levels were significantly lower in survivors (49 (82) mg/L) compared to non-survivors (112 (102) mg/L; *p* < 0.001). Serum creatinine levels were also significantly lower in survivors (0.9 (0.7) mg/dL) than in non-survivors (1.3 (1.2) mg/dL; *p* < 0.001).

The mean prognostic nutritional index was 39 ± 7.5 in the survivor group and 34 ± 7.3 in the non-survivor group. This difference was statistically significant (*p* < 0.001).

A correlation analysis revealed that the prognostic nutritional index was positively correlated with the platelet count (r = 0.15, *p* = 0.005). It was negatively correlated with age (r = −0.25, *p* < 0.001), mean platelet volume (r = −0.16, *p* = 0.002), C-reactive protein (r = −0.21, *p* < 0.001), and serum creatinine (r = −0.14, *p* = 0.01).

**Figure 2:**
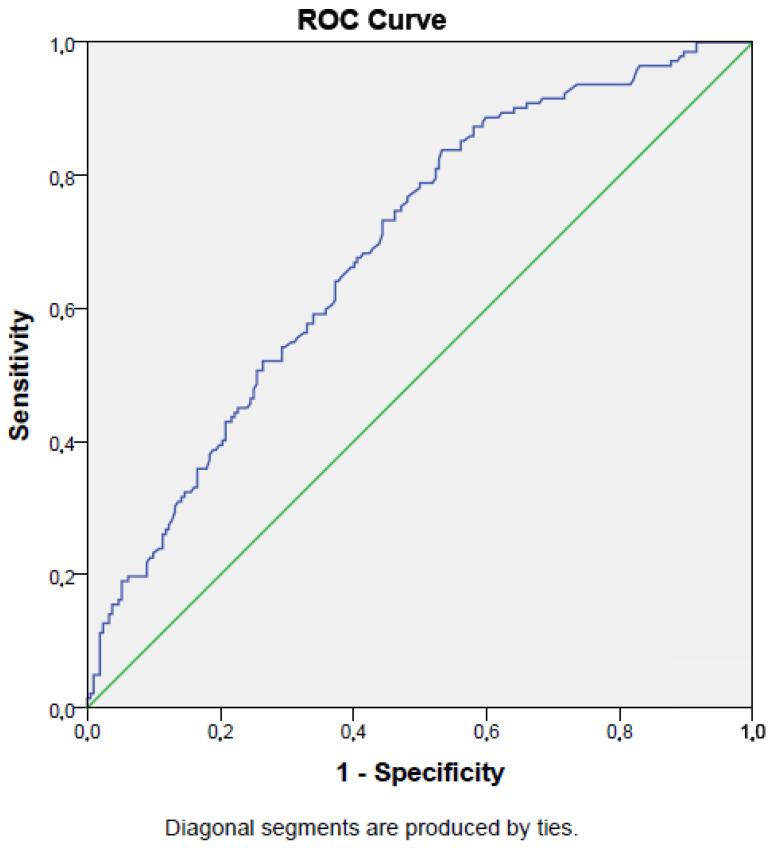
ROC curve of prognostic nutritional index in detecting survival of intensive care unit population.

The sensitivity and specificity of the prognostic nutritional index for predicting mortality (cut-off value < 34.2%) were 73% and 56%, respectively (AUC = 0.69, *p* < 0.001; 95% CI: 0.64–0.74). Figure 2 presents the ROC curve for the PNI in detecting mortality.

A binary logistic regression analysis showed that the prognostic nutritional index was an independent predictor of mortality in ICU patients, even after adjusting for age, mean platelet volume, platelet count, C-reactive protein, and serum creatinine. Each unit increase in prognostic nutritional index reduced the risk of mortality by 6% (*p* < 0.001; OR: 0.94, 95% CI: 0.90–0.97). Table 2 shows the odds ratios of the study variables.

## 4. Discussion

The main outcomes of the present study were as follows:

(a) The prognostic nutritional index was significantly lower in deceased patients compared to survivors in the intensive care unit;
(b) Prognostic nutritional index was significantly correlated with serum creatinine, CRP, PLT, MPV, and age in ICU patients;
(c) Prognostic nutritional index demonstrated moderate sensitivity and fair specificity in predicting mortality among ICU patients;
(d) Prognostic nutritional index was an independent risk factor for mortality in patients treated in the ICU.

We also found elevated CRP levels in deceased patients compared to survivors, which is consistent with findings in the literature. CRP is an acute-phase protein produced by the liver in response to inflammation, infection, or tissue injury, making it a reliable marker of systemic inflammation. Elevated CRP levels in deceased ICU patients suggest a more severe inflammatory response, potentially due to infections (e.g., sepsis), organ dysfunction, or other critical conditions. Persistent or excessively high CRP levels are associated with poorer prognosis, as unchecked inflammation may lead to multi-organ failure, septic shock, or worsening of comorbidities [13]. Conversely, survivors typically exhibit a more controlled or resolving inflammatory response, correlating with lower CRP levels and improved outcomes. Therefore, monitoring CRP can assist clinicians in assessing disease severity, guiding therapeutic decisions, and predicting prognosis.

Another important finding was the significantly lower PNI in deceased ICU patients. This underscores the importance of nutritional and immune status in determining outcomes. The PNI, calculated using serum albumin and total lymphocyte count, reflects both nutritional reserves and immune function. A lower PNI indicates malnutrition and immunosuppression, both of which contribute to poor outcomes [14]. Malnutrition in critically ill patients hinders tissue repair, immune defense, and stress response, increasing vulnerability to infections, delayed wound healing, and organ failure [15]. A low lymphocyte count also reflects a compromised immune system, elevating the risk of sepsis and related complications [16]. In contrast, higher PNI values suggest better nutrition and immune function, supporting recovery and survival.

Previous studies have similarly highlighted PNI’s prognostic value. Peng et al. reported that a low PNI predicted worse outcomes in patients with chronic obstructive pulmonary disease [17]. Additionally, reduced PNI levels have been associated with higher mortality in sepsis patients [18]. Accordingly, we observed decreased prognostic nutritional index levels in deceased ICU patients compared to survivors. This finding suggests that the early identification of nutritional deficits and immune dysfunction using the PNI may be crucial for implementing targeted interventions—such as nutritional support and immunomodulation—to improve outcomes in ICU patients.

Platelets are essential for blood clotting and play a vital role in preventing excessive bleeding. In addition, they contribute to the body’s immune defense and tissue repair. A decrease in platelet count is a common complication among ICU patients and is frequently associated with poorer outcomes [19]. In deceased ICU patients, low platelet counts may serve as a marker of severe illness, such as disseminated intravascular coagulation (DIC), sepsis, and multi-organ failure—conditions that are common in critically ill individuals and often precede death. Thrombocytopenia can result from various causes, including systemic infections, which may trigger an excessive immune response that leads to increased platelet destruction or consumption [20]. Moreover, liver dysfunction, which is common in critically ill patients, can impair platelet production, as the liver plays a crucial role in synthesizing thrombopoietin, a hormone essential for platelet formation [21]. In contrast, survivors generally maintain higher platelet counts, suggesting a better ability to manage bleeding risk and immune responses, as well as a less severe overall disease course. Monitoring platelet counts in intensive care unit patients can serve as an important prognostic marker, helping clinicians identify those at higher risk of complications and mortality. Numerous studies in the literature have reported that a low platelet count is a predictor of mortality in the intensive care unit population [22,23]. In accordance with the literature, we found reduced platelet counts in deceased patients compared to survivors.

The present study also showed that the mean platelet volume was lower in deceased intensive care unit patients compared to survivors. The mean platelet volume is a measure of the average size of platelets in the blood, with larger platelets generally being more active and possessing greater hemostatic potential [24]. Interestingly, the mean platelet volume has been proposed as a marker of inflammation in various conditions, including infections [25], and vertebral disc disease [26]. All of these conditions are characterized with inflammation as the deceased subjects in intensive care. Additionally, the mean platelet volume is often used as an indirect marker of platelet production and activity, and changes in the mean platelet volume can indicate alterations in the platelet function or bone marrow response. The decreased mean platelet volume in deceased intensive care unit patients in the present study may suggest that these patients had impaired bone marrow production of platelets or a diminished ability to release larger, more active platelets into circulation. This could be a consequence of severe systemic conditions such as sepsis, multi-organ failure, or other critical illnesses that suppress bone marrow activity or lead to increased platelet destruction. Another possible explanation could be that the mean platelet volume may decrease due to the consumption of larger platelets because of inflammation and remaining smaller platelets in the circulation [27]. On the other hand, intensive care unit survivors with higher mean platelet volume levels may have a better capacity for platelet production and function, reflecting more robust bone marrow activity or a more controlled inflammatory response. Larger platelets are typically more reactive, which could offer better protection against bleeding and aid in tissue repair and immune defense. The increased mean platelet volume in survived patients and decreased mean platelet volume in deceased subjects in the present study highlights the importance of monitoring the mean platelet volume as part of a broader assessment of platelet function and overall health status in intensive care unit patients.

The prognostic nutritional index was correlated with various study variables in the present report. These correlations may be a consequence of interaction between nutritional status, inflammation, age, and organ function in critically ill patients. We showed that prognostic nutritional index was correlated with PLT in the study cohort. This finding suggests that better nutritional and immune status is associated with higher platelet levels in intensive care unit patients. Platelets play a crucial role not only in coagulation but also in immune responses and tissue repair [28]. Higher prognostic nutritional index values reflect better nutritional reserves and immune function, which may promote more intact bone marrow activity and platelet production. This association implies that malnourished patients (with lower prognostic nutritional index) might also have lower platelet counts, which could contribute to an increased risk of poor outcomes in the intensive care unit setting.

The present study revealed an inverse correlation between the prognostic nutritional index and the age of the patients. That means older intensive care unit patients tend to have lower prognostic nutritional index values. This is likely due to the fact that aging is often accompanied by sarcopenia, decreased nutrient intake, and a general decline in immune function [29,30,31]. Older individuals also have a higher likelihood of chronic diseases that can affect both nutritional and immune status. The inverse correlation between age and prognostic nutritional index in the present study warrants the necessity for giving particular attention to the nutritional needs of older intensive care unit patients, as they may be at higher risk for malnutrition and related complications.

We also reported an inverse correlation between the prognostic nutritional index and C-reactive protein. The negative correlation between prognostic nutritional index and C-reactive protein suggests that patients with higher levels of systemic inflammation tend to have lower prognostic nutritional index values. Inflammation often leads to a catabolic state, where protein breakdown exceeds synthesis, resulting in malnutrition and a reduction in immune competence [32]. Elevated C-reactive protein levels reflect a more severe inflammatory response, which can deplete nutritional reserves and worsen patient outcomes. An inverse correlation between C-reactive protein and prognostic nutritional index in the present work emphasizes the interaction between inflammation and nutrition in critically ill patients and the importance of managing both aspects to improve patient outcome.

The present study also revealed an inverse correlation between the prognostic nutritional index and serum creatinine levels, which suggests that patients with poorer nutritional status may have impaired renal function. Elevated serum creatinine is a marker of kidney dysfunction, which can contribute to malnutrition through fluid imbalances, reduced nutrient absorption, and metabolic disturbances [33]. For instance, acute kidney injury is common in critically ill patients and can exacerbate malnutrition by increasing catabolism and reducing dietary intake [34]. The negative correlation between the prognostic nutritional index and serum creatinine, as reported in the present study, suggests that nutritional support might be required for intensive care unit patients with kidney dysfunction to improve their prognosis.

The sensitivity and specificity of the prognostic nutritional index in detecting mortality in intensive care unit patients were not low in the present work. A sensitivity of 73% and specificity of 56% for the prognostic nutritional index were demonstrated in mortality detection. The prognostic nutritional index can correctly select 73% of patients who died in intensive care unit when the cut-off value is lower than 34.2%. This represents moderately high sensitivity, meaning the prognostic nutritional index is relatively effective in identifying critically ill patients who are likely to have poor outcomes. Similar reports have been published in the literature. Wang et al. found that the prognostic nutritional index had 73% sensitivity and 53% specificity in detecting the survival of the patients with hepatocellular carcinoma [35]. The usefulness of the prognostic nutritional index has been shown in another study, which reported a 60% sensitivity and 83% specificity of the prognostic nutritional index in detecting the survival of rectal cancer patients [36]. In addition, a meta-analysis revealed that the prognostic nutritional index had 61% sensitivity and 60% specificity in detecting the mortality of the patients with esophageal cancer [37]. These data suggest that the prognostic nutritional index could be useful in detecting the mortality of the conditions characterized with chronic inflammation. The sensitivity and specificity of the prognostic nutritional index in detecting mortality in the present study was comparable to the literature data.

We also demonstrated that the prognostic nutritional index was an independent risk factor for mortality in intensive care unit patients. This finding emphasizes the critical role of nutritional status in determining patient outcomes in intensive care. The effects of the prognostic nutritional index on the survival of the patients in intensive care unit did not rely on the influence of age, mean platelet volume, PLT, C-reactive protein, or serum creatinine. Thus, the argument that the prognostic nutritional index serves as a vital indicator of a patient’s overall health and prognosis in the intensive care unit, specifically reflecting the nutritional and immune status that can significantly impact recovery and survival, is strengthened. Similar findings has been reported in the literature. Jiang et al. reported that the prognostic nutritional index was a risk factor of survival in patients with esophagus cancer with a 1.51 odds ratio [37]. Moreover, the hazard ratio of a low prognostic nutritional index was 1.3 in the survival of colorectal cancer patients [38]. Similarly, low hazard ratio values have been reported in patients with hip fracture who have higher PNI values [39]. These data suggest the findings of the present work.

Elevated NLR has been reported in the present study in deceased subjects compared to the survivors. The literature contains many similar reports. For example, Regolo et al. found that NLR was an independent prognostic indicator in patients with a COVID-19 infection [40]. Moreover, it was suggested as a useful tool in differentiating bacterial and viral infections in ICU populations [41]. In accordance with the literature data, we reported higher NLR values in deceased ICU patients compared to the survived patients.

Several limitations may limit the accuracy of our findings in the present study. First, the study was retrospective, meaning we just found a simple association rather than causal relationship between the prognostic nutritional index and mortality of intensive care unit patients. Second, the study cohort was relatively small. Third, the single-center nature of the work may limit the globalization of the study findings. However, importantly, the present study reported the prognostic nutritional index as an independent risk factor of mortality in the intensive care unit setting.

## 5. Conclusions

In conclusions, these findings highlight the critical importance of nutritional assessment in the intensive care unit and establishes prognostic nutritional index as a valuable prognostic tool. Given its independent association with mortality risk, physicians should integrate regular prognostic nutritional index evaluations into their clinical practice to identify at-risk patients early. Thus, timely nutritional interventions and the improvement of the management of critically ill patients ultimately enhanced patient outcomes and survival rates in the intensive care unit setting and could therefore be implemented.

## Figures and Tables

**Table 1:** Summary of the general characteristics and laboratory data of the study population.

		Survived Group (*n* = 142)	Deceased Group (*n* = 212)	*p*
Gender	Men (n, (%))	82 (58)	130 (61)	0.50
	Women (n, (%))	60 (42)	82 (39)	
		Median (IQR)		
Age (years)		68 (27)	73 (22)	**<0.001**
Serum albumin (g/dL)		3.2 (0.8)	2.8 (0.7)	**<0.001**
WBC (×10^3^/mm^3^)		11.5 (8.8)	12.6 (10)	0.46
neu (×10^3^/mm^3^)		9.5 (8.2)	10.5 (9)	0.19
lym (×10^3^/mm^3^)		1.12 (0.8)	1(0.9)	**0.02**
mono (×10^3^/mm^3^)		0.6 (0.4)	0.6 (0.5)	0.30
RDW (%)		17 (4)	16 (4)	0.40
PLT (×10^3^/mm^3^)		217 (115)	199 (157)	**0.002**
PDW (%)		17 (4)	17 (6)	0.09
MPV (fL)		9 (3)	8 (2.9)	**<0.001**
PG (mg/dL)		133 (75)	143 (85)	0.06
CRP (mg/L)		49 (82)	112 (102)	**<0.001**
Serum creatinine (mg/dL)		0.9 (0.7)	1.3 (1.2)	**<0.001**
Length of ICU stay (days)		3 (0–118)	5 (0–97)	**0.004**
NLR (%)		8.4 (1.9–15.7)	10.4 (4–32)	**0.01**
		Mean ± SD		
Hb (g/dL)		12 ± 2.5	13 ± 2.4	0.48
Htc (%)		36 ± 8	39 ± 7	0.56
PNI (%)		39 ± 7.5	34 ± 7.3	**<0.001**

**Table 2:** Odds ratios of the variables included in logistic regression analysis.

		*p*	OR	95% CI
prognostic nutritional index		<0.001	0.94	0.90–0.97
age		0.001	1.03	1.01–1.04
mean platelet volume		<0.001	1.31	1.16–1.48
Platelet count		0.93	1	1–1.002
C-reactive protein		0.001	1.01	1.002–1.09
Serum creatinine		0.36	1.1	1.02–1.33

## Data Availability

The data presented in this study are available on request from the corresponding author due to ethical issues (Institutional ethics committee does not allow datasets to be publicly available).

## Abbreviations

The following abbreviations are used in this manuscript:

WBC	White blood cell count
neu	Neutrophil count
lym	Lymphocyte count
mono	Monocyte count
RDW	Red cell distribution width
PLT	Platelet count
Hb	Hemoglobin
Htc	Hematocrit
PDW	Platelet distribution width
MPV	Mean platelet volume
PG	Plasma glucose
CRP	c-reactive protein
PNI	Prognostic nutritional index
